# Alternating EM algorithm for a bilinear model in isoform quantification from RNA-seq data

**DOI:** 10.1093/bioinformatics/btz640

**Published:** 2019-08-10

**Authors:** Wenjiang Deng, Tian Mou, Krishna R Kalari, Nifang Niu, Liewei Wang, Yudi Pawitan, Trung Nghia Vu

**Affiliations:** Department of Medical Epidemiology and Biostatistics, Karolinska Institutet, Stockholm 17177, Sweden; Department of Medical Epidemiology and Biostatistics, Karolinska Institutet, Stockholm 17177, Sweden; Department of Health Sciences Research, MN 55905, USA; Department of Molecular Pharmacology and Experimental Therapeutics, Mayo Clinic, Rochester, MN 55905, USA; Department of Molecular Pharmacology and Experimental Therapeutics, Mayo Clinic, Rochester, MN 55905, USA; Department of Medical Epidemiology and Biostatistics, Karolinska Institutet, Stockholm 17177, Sweden; Department of Medical Epidemiology and Biostatistics, Karolinska Institutet, Stockholm 17177, Sweden

## Abstract

**Motivation:**

Estimation of isoform-level gene expression from RNA-seq data depends on simplifying assumptions, such as uniform read distribution, that are easily violated in real data. Such violations typically lead to biased estimates. Most existing methods provide bias correction step(s), which is based on biological considerations—such as GC content—and applied in single samples separately. The main problem is that not all biases are known.

**Results:**

We have developed a novel method called XAEM based on a more flexible and robust statistical model. Existing methods are essentially based on a linear model *Xβ*, where the design matrix *X* is known and is computed based on the simplifying assumptions. In contrast XAEM considers *Xβ* as a bilinear model with both *X* and *β* unknown. Joint estimation of *X* and *β* is made possible by a simultaneous analysis of multi-sample RNA-seq data. Compared to existing methods, XAEM automatically performs empirical correction of potentially unknown biases. We use an alternating expectation-maximization (AEM) algorithm, alternating between estimation of *X* and *β*. For speed XAEM utilizes quasi-mapping for read alignment, thus leading to a fast algorithm. Overall XAEM performs favorably compared to recent advanced methods. For simulated datasets, XAEM obtains higher accuracy for multiple-isoform genes. In a differential-expression analysis of a real single-cell RNA-seq dataset, XAEM achieves substantially better rediscovery rates in independent validation sets.

**Availability and implementation:**

The method and pipeline are implemented as a tool and freely available for use at http://fafner.meb.ki.se/biostatwiki/xaem/.

**Supplementary information:**

Supplementary data are available at *Bioinformatics* online.

## 1 Introduction

Alternative splicing is a regulated process where inclusion and exclusion of exons from the same gene produce multiple transcripts. Alternative gene transcripts can also arise from alternative start or stop locations. Transcripts originating from the same gene have mostly similar sequences, but can have distinct biological functions (Christofk *et al.*, 2008; Steinberg, 2008). Thus, variability in alternative transcription greatly increases the biodiversity and functional complexity in higher eukaryotes. Alternative transcripts are also known as isoforms, so for convenience we use these terms interchangeably. Quantifying gene expression at isoform level is an important step in studies of molecular processes and cancer research, e.g. tumor-subtype classification and stage tracking in cancer progression (Weinstein *et al.*, 2013). Therefore, development of computational methods to estimate isoform-level expression accurately and efficiently is of great importance.

All methods of isoform expression estimation start with basic simplifying assumptions, such as uniform read distribution (Jiang and Wong, 2009; Mortazavi *et al.*, 2008; Srivastava *et al.*, 2016). In reality such assumptions are easy to violate: e.g. the read distribution can be non-uniform due to a number of experimental and technical biases. When generating the cDNA fragments from RNA transcripts, there is a possibility of a preference to the 5′ or 3′, which produces more reads at the start/end of a fragment. Other factors such as GC bias and sequence-specific biases may also negatively affect the quantification. The accuracy of isoform expression inference can deteriorate if the biases are not taken into account. So bias is a well-known issue in isoform quantification.

In fact most isoform quantification methods employ bias correction steps. Salmon (Patro *et al.*, 2017) implements a two-step procedure, with a sample specific model to correct bias in the second step. Cufflinks starts with a uniformity assumption, but provides a likelihood-based approach for bias correction (Roberts *et al.*, 2011; Trapnell *et al.*, 2010). The correction in Kallisto (Bray *et al.*, 2016) is similar to that in Cufflinks, while it measures the sequence-specific bias from the first 1 million reads. In our previous work (Suo *et al.*, 2014), we demonstrated that there is a high correlation of relative abundances of reads between samples, suggesting that summarizing distribution information across samples could correct for the underlying non-uniform read distribution. All these methods attempt to identify the sources of biases explicitly and provide specific corrections for them. The main problem is that not all biases are known, and there is still no general approach that can adapt to unknown biases in RNA-seq data.

Read mapping or alignment is also a source of problems in isoform quantification using RNA-seq data. Due to the nature of isoforms, an aligner must take into account the splicing mechanism in alignment of read. A wide range of splice-aware aligners have been developed for this purpose, e.g. Tophat (Trapnell *et al.*, 2009), STAR (Dobin *et al.*, 2013) and HISAT (Kim *et al.*, 2015). Although these tools facilitate the read alignment and significantly reduce memory usage, the alignment step remains time consuming, especially when they report optimal alignments with comprehensive information, e.g. CIGAR string.

Recently, a variety of methods have been used to reduce the burden of the read alignment step. These approaches can increase the speed by orders of magnitude, while achieving similar estimation accuracy as the traditional method. For example, Sailfish (Patro *et al.*, 2014) introduces the concept of *lightweight-alignment*, which supports the idea that precise alignment is not necessary to assign the potential origin of a sequencing read. Similarly, Kallisto (Bray *et al.*, 2016) introduces a *pseudo-alignment* to skip the read alignment step. Salmon (Patro *et al.*, 2017) implements a *quasi-mapping* to allow fast and accurate alignment of fragments to the reference transcriptome. It makes use of a hash table and suffix array (SA) to locate the potential transcript of origin of a sequenced read. This quasi-mapping is also implemented as an independent mapper, i.e. Rapmap, and applied to the updated versions of Sailfish (Srivastava *et al.*, 2016).

To address these issues, we have developed a novel method called XAEM for isoform expression estimation based on a more flexible and robust statistical model. Existing methods are essentially based on a linear model *Xβ*, with known design matrix *X* derived using simplifying assumptions such as uniform read distribution, and the unknown isoform abundance *β* is estimated using the EM algorithm. Explicit bias correction is then performed as an extra step, and applied sample-by-sample, using separate considerations. In contrast, XAEM considers *Xβ* as a bilinear model with both *X* and *β* unknown. Joint estimation of *X* and *β* is made possible by a simultaneous analysis of multi-sample RNA-seq data. Compared to existing methods, the bilinear model automatically performs empirical correction of potentially unknown biases. We use an alternating expectation-maximization (AEM) algorithm, alternating between estimation of *X* and *β*. For speed XAEM utilizes the quasi-mapping approach for ultra-fast read mapping. We compare XAEM with several state-of-the-art quantification tools including Salmon (Patro *et al.*, 2017), Kallisto (Bray *et al.*, 2016), Sailfish (Srivastava *et al.*, 2016) and Cufflinks (Trapnell *et al.*, 2010). The comparison is performed on both simulated and real RNA-seq datasets. To illustrate XAEM’s ability to deal with potentially unknown biases, the real data are single-cell (sc)RNA-seq data from breast-cancer cells. The results show that XAEM achieves overall better performances in both quantification of isoform-level expression and detection of differentially expressed isoforms.

## 2 Materials and methods

### 2.1 General isoform quantification model

A major problem in conventional isoform quantification is how to quantify reads that map to an exon shared by different isoforms. The simple illustration in Supplementary Figure S1 shows three single-end reads mapped to exon1 shared by three transcripts: tx1, tx2 and tx4. It is not obvious which transcript these reads originate from. In this context we also use ’isoform’ which refers to alternative gene transcript. We extend the idea of the exon-sharing to a more general concept: sequence-sharing. Due to the sequence similarity, one fragment can map to several reference sequences. Thus, any highly similar sequence could lead to exon-sharing, but the exons are not necessarily within the same gene. We can call the similar sequence as *quasi-exon*. Thus throughout the text, the term exon indicates quasi-exon. In order to describe exon-sharing in isoform expression estimation, we use the concept of ‘equivalence class’ (eqclass) from a recent paper (Srivastava *et al.*, 2016). An equivalence class is generated from reads mapping statistics, so it does not need to have direct biological meaning, especially for paired-end data. For single-end data, the simplest interpretation of an eqclass is as a list of transcripts sharing the same exon, so the class may represent the exon.

We first summarize the number of reads that map to different eqclasses using Rapmap (Srivastava *et al.*, 2016) from an input RNA-seq sample. Let *y_j_* be the number of reads (read-count) that are mapped to eqclass *j*. For a specific set of eqclasses *J* with *T* transcripts, let *β_t_* be the expression level of transcript *t*. The key statistical problem is to estimate transcript abundances *β_t_* from the read-count data $y=\{y_{j},j=1,\ldots ,J\}$. By adding up the contribution of multiple transcripts, we model the expected number of reads in eqclass *j* as

(1) $$\mu_{j}=w\sum_{t}a_{\mathrm{jt}}L_{t}p_{\mathrm{jt}}\beta_{t}\equiv \sum_{t}x_{\mathrm{jt}}\beta_{t},$$

which, in matrix notation, can be written as

(2) μ=Xβ,

where $x_{\mathrm{jt}}\equiv wa_{\mathrm{jt}}L_{t}p_{\mathrm{jt}}$. Here *w* refers to the total number of mapped reads divided by 10^9^ (per kilobase per million reads), *a_jt_* is the isoform-specific bias/non-uniformity effect, *L_t_* is effective length and *p_jt_* is the proportion of reads in eqclass *j* under uniform distribution. For identifiability we set $\sum_{j}x_{\mathrm{jt}}\equiv 1$ for every transcript *t*. It is conventionally assumed that *y_j_* has Poisson distribution with mean *μ_j_*.

In general both *X* and *β* in equation (2) are unknown parameters, so we have a bilinear model. Under the uniform read distribution assumption, we have $a_{\mathrm{jt}}\equiv 1$, so (1) becomes

(3) $$\mu_{j}\equiv w\sum_{t}L_{t}p_{\mathrm{jt}}\beta_{t}$$

The simple model (3) is either explicitly or implicitly used by most existing RNAseq quantification tools (Jiang and Wong, 2009; Mortazavi *et al.*, 2008; Srivastava *et al.*, 2016). We also use it to generate the starting value of the design matrix *X*, as described in the next section.

### 2.2 Construction of the starting design matrix *X*

The starting value of *X* in the XAEM algorithm is derived by assuming the uniform read distribution. From equation (3), *X* contains three components: (i) a list of transcripts connected due to exon-sharing, (ii) a set of exons shared by these transcripts and (iii) the proportion of read counts from the eqclasses that contribute to the total reads of each transcript. As an illustration, suppose the corresponding design matrix for Supplementary Figure S1 is given by (4), which contains four transcripts: tx1, tx2, tx3 and tx4 (columns) and three eqclasses (rows). The values *x_jt_* in the matrix indicate the proportion of reads originating from eqclass *j* for transcript *t*. Note that the sum of proportions for each transcript $\sum_{j}x_{\mathrm{jt}}$ is equal to 1.

(4) $$X=\left(\begin{array}{cccc} 0.02 & 0.12 & 0 & 0.15\\ 0.85 & 0.88 & 1 & 0\\ 0.13 & 0 & 0 & 0.85 \end{array}\right).$$

We now describe in detail how to construct the design matrix in (4) using simulated RNA-seq data. The full set of design matrices for all transcripts in the transcriptome can be generated in the same way.

#### 2.2.1 Construction of transcription clusters (TCs)

The eqclass shows the connection between a set of transcripts sharing the same exon. To investigate this connection, we use Polyester (Frazee *et al.*, 2015) to generate simulated reads for each transcript across the transcriptome. For example, we first simulate sample1 for transcript tx1. In sample1, we set tx1 to be the only transcript expressed, and the read count is assigned a large number *L*. In practice, we used *L *=* *2× transcript length, but with a minimum of 1000. For this simulated data, reads with uniform distribution are generated with base error rate set to 0.005. We then use Rapmap (Srivastava *et al.*, 2016) to align the reads to the reference transcriptome and summarize the mapping results into a matrix. We denote this matrix as *transcript response profile* (TRP), which defines a set of transcripts sharing exons with the source transcript, which is tx1 in this example.

Table 1 shows an example of TRP of tx1. There are four transcripts sharing exons with tx1 by three eqclasses. The three rows in Table 1 represent the three eqclasses and the values indicate the proportion of reads originating from each eqclass. From the table, we observe that other than tx1, which is the only transcript that is supposed to be expressed, there are four extra transcripts having mapped reads due to exon-sharing. This information is useful to capture the exon-sharing between different transcripts. When there is more than one transcript in the TRP, we denote these transcripts as *transcript neighbors* (TNs). To remove the false positives, the neighbors with reads less than a threshold *H* (e.g. 2.5% of total reads from the source transcript) are filtered out. Thus, in this example tx5 is discarded, and tx1 has three remaining neighbors: tx2, tx3 and tx4. We also evaluate the effect when we set the *H* as 0, which means all the neighbors are kept in a TRP. As shown in Supplementary Table S1, the performance is almost the same with *H *=* *2.5%. However, we would still suggest to use a threshold to filter false positive neighbors in the TRPs.

**Table 1.** An example of transcript response profile (TRP) matrix for tx1 based on simulated sample1

Eqclass	tx1	tx2	tx3	tx4	tx5
eqclass1	20	20	0	20	20
eqclass2	850	850	850	0	0
eqclass3	130	0	0	130	0
Sum	1000	870	850	150	20

*Note*: In this sample1 we set only tx1 expressed and we use Rapmap for read mapping. Next we summarize the read alignment results into the TRP matrix. The exon-sharing is presented by equivalence class (eqclass) as the rows in the matrix. Transcript tx5 is filtered out because it fails the false positive threshold of H = 2.5%.

**Fig. 1.**
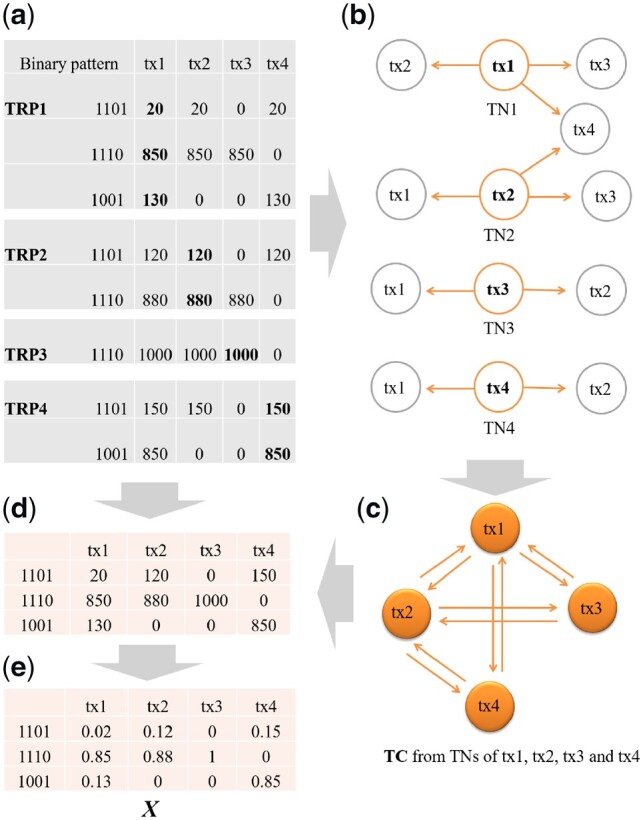
Steps to construct the starting design matrix *X*. (**a**) TRPs of tx1, tx2, tx3 and tx4, and the summary of binary occupancy patterns from the TRPs. Transcript tx5 does not pass the filtering (H = 2.5%) and is filtered out from TRP1. In each binary pattern, digit 1 means there are reads originating from an eqclass, and 0 otherwise. For example, there are three eqclasses in TRP1: eqclass1, eqclass2 and eqclass3. For eq1 the binary pattern is 1101, which means three transcripts, i.e. tx1, tx2 and tx4 have reads from eq1. (**b**) Transcript neighbors (TNs) for tx1 to tx4. (**c**) Illustration of construction of transcription cluster (TC) from the TNs. We first collect the TNs of tx1, tx2, tx3 and tx4, and then add the connections between transcripts into the TC. For example, from TN1, we add the connection of tx1-tx2, tx1-tx3 and tx1-tx4. In the end, a TC would contain all connections between transcripts sharing exons. (**d**) The unique set of binary patterns are kept, so three unique patterns remain: 1101, 1001, 1110. We then fill in the read counts from each source TRP. For example, for pattern 1101, in TRP1 the read count is 20 for tx1, in TRP2 the read count is 120 for tx2 and in TRP4 the read count is 150 for tx4. (**e**) The total reads of each transcript in (d) are standardized to sum to 1 to create the starting design matrix *X*

Similarly, we generate TRP and TN for each transcript (Fig. 1a and b). To expand the list of transcripts that have connections, we group the TRPs of connected transcripts together as *transcription clusters* (TCs). As shown in Figure 1c, starting with tx1, we integrate the TNs of tx1, tx2, tx3, tx4 and summarize the connections into one TC. Thus each TC defines one unique design matrix with the list of mutually connected transcripts.

#### 2.2.2 Computing *X* for each TC

We combine the information of TC and TRPs to create a design matrix *X*. Particularly, we summarize the reads from the TRPs into one table (Fig. 1d). In this table we convert the names of eqclasses to a binary occupancy pattern. The digits 1 and 0 indicate whether a transcript has supporting reads from an eqclass or not. We then keep the final set of binary patterns as the eqclasses in the rows of the *X* matrix. The original read counts of each transcript originated from different eqclasses, which are highlighted by the bold numbers in Figure 1a, are transferred to the corresponding positions in the matrix (Fig. 1d). Finally the total reads for each transcript is standardized to sum to 1, which is shown in Figure 1e. The final *X* matrix is exactly the design matrix in formula (4).

#### 2.2.3 Merging paralogs

Paralogs are isoforms that have high similarity in their sequences. Paralogs complicate the quantification of isoform expression due to the difficulty in distinguishing them from each other. If there are paralogs in *X* matrix, the columns of the corresponding transcripts can be extremely similar to each other, thus making the *X* matrix singular and the *β* parameter non-identifiable. To deal with this issue, we implement the k-means clustering to combine the paralogs into one transcript. Theoretically, exact paralogs will produce zero singular values, so the number of clusters *k* is the number of non-zero singular values in the singular-value decomposition of *X*. But in practice, we may get values very close to zero. As shown in Supplementary Section S2, based on our numerical studies, we set *k* as the number of singular values greater than 0.02. An illustrative example of the merging process can be seen in the Supplementary Section S3. The final set of processed *X* matrices is used as the starting *X* in the model (2).

### 2.3 Estimation using the alternating EM algorithm

**Fig. 2.**
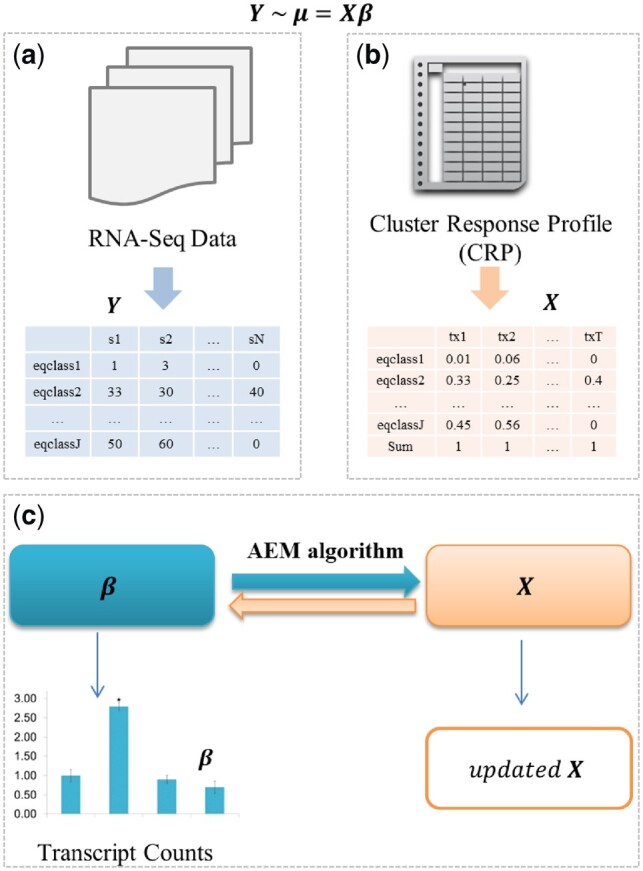
Pipeline of XAEM for isoform expression estimation. (**a**) Construction of *Y* matrix from the input RNA-seq data of multiple samples. (**b**) An example of *X* or design matrix as the input of the model. (**c**) The step to estimate *X* and *β* iteratively using the AEM algorithm. In the last step, XAEM outputs the updated design matrix *X* and isoform expression *β*

Figure 2 shows the workflow of XAEM for isoform expression estimation. First, we summarize the observed read counts *y_jn_* from input RNA-seq sample *n*. The RNA-seq reads are mapped to the reference transcriptome using the quasi-mapping algorithm. We extract the list of eqclasses and their read counts *y_jn_*, then match them in accordance with the eqclasses in the design matrices. To be concise we simply denote *y_jn_* as *y_j_* in the following text. We then summarize the read counts *y_j_* across multiple samples and construct matrix *Y* as described in Figure 2a. Next, joint estimation of *X* and *β* is done using the alternating expectation-maximization (AEM) algorithm, as shown in Figure 2c. Given the starting value of *X*, the transcript abundance *β_t_* can be estimated from the model in formula (5):

(5) $$\mu_{j}=\sum_{t}x_{\mathrm{jt}}\beta_{t}$$

Thus, the estimation can be performed iteratively as follows:

A. Given *x_jt_*, estimate *β_t_* for multiple samples using (5)

B. Given *β_t_*, estimate *x_jt_* using model:

(6) $$\mu_{j}=\sum_{t}\beta_{t}x_{\mathrm{jt}}$$

C. Iterate steps A and B until convergence.

Specifically, in step A, given *x_jt_* to estimate *β_t_*, we calculate the value of *β_t_* using

(7) $$\beta_{t}^{i+1}=\beta_{0}+\sum_{j}y_{j}\frac{e^{\ell_{j}^{i}}}{\sum_{t\in j}e^{\ell_{t}^{i}}}$$

where

(8) $$\ell_{j}^{i}=\psi (\beta_{0}+\beta_{t}^{i})-\psi (\sum_{t\in j}\beta_{0}+\beta_{t}^{i}),$$

and ψ(*) is the digamma function used in the E-step of the modified EM algorithm (Nariai *et al.*, 2013); *β*_0_ is a starting estimate for *β_t_*, e.g. the mean read counts among *T* transcripts. In step B, for the updated *X* we standardize *x_jt_* for each eqclass to calculate the proportion of read counts from observed counts *y_j_*. The parameter *β_t_* is then re-estimated using the proportion multiplied by *y_j_*. The iteration procedure is considered to be converged when both *x_jt_* and *β_t_* have less than 1% difference between successive iterations. The 1% threshold is for maximal differences across the matrix elements, and if a matrix element is <0.01 then the threshold is set to 1% of 0.01.

It is worth noting here that XAEM does not individually deal with different types of biases. The effect of biases generally changes the proportion of reads to transcripts in the design matrix. By updating the design matrix *X* during the isoform quantification in AEM, XAEM automatically adjusts the design matrix for bias correction using the variation information from multiple samples. We have tested the robustness of the *X* matrix using different tissue data from the genotype-tissue expression (GTEx) project (Lonsdale *et al.*, 2013). As shown in Supplementary Sections S4 and S5, the performance of XAEM is robust with respect to the heterogeneity of input samples.

### 2.4 Datasets

#### 2.4.1 Simulated RNA-seq data

RNA-seq simulation is commonly used for the comparison of estimation accuracy among different tools. To investigate the estimation performance in a situation where the true expression levels are known, we use a simulator named Polyester (Frazee *et al.*, 2015) to generate synthetic datasets. To mimic a real RNA-seq dataset, we use scRNA-seq data from a human colon cancer cell line HCT116 (Wu *et al.*, 2014) as the source of true *β* values. We first derive the isoform-level expression using Sailfish (Srivastava *et al.*, 2016) and implement the four-parameter beta-Poisson model to fit isoform abundance (Vu *et al.*, 2016). Next, we collect the well-fitted models (Monte-Carlo *P*-value >0.05) as the baseline distribution for isoform expression. The models are assigned to isoforms in the transcriptome. For each isoform, the expression level across samples is generated from the assigned beta-Poisson model.

Read counts are calculated by scaling the expression values to the predefined library size of samples. The library size are also derived from the HCT116 cell line, which ranges from 1 to 2.8 million. We notice that more than 70% of isoforms are unexpressed. We first compare the performance of XAEM and Salmon using this original data. The comparison shows that XAEM still has higher accuracy for paralogs (Supplementary Table S2). But in this comparison only 2607 multiple isoforms are used, which cover only 8% of all multiple isoforms existing in the transcriptome (total 31 950). To simulate a more heterogeneous dataset, we assign the read counts from expressed isoforms to those unexpressed. The aims for this re-distribution are: (i) creating a more heterogeneous and comprehensive dataset for the comparison; (ii) preserving the distribution of read counts and expression patterns as in the original cell line. Note that the re-distribution procedure is conceptually related to a non-zero prior belief of transcript expression, and is thus theoretically justifiable. Furthermore, we re-distribute the read counts randomly to those unexpressed isoforms, and do not pick specific isoforms for the re-distribution. Polyester allows users to specify positional bias so that the read distribution can be uniform or non-uniform. In order to compare the quantification accuracy in different scenarios, we simulate 100 samples with uniform distribution and another 100 samples with non-uniform distribution. Paired-end reads are simulated with read length 100 bp, mean fragment length of 250 bp and standard deviation of fragment lengths of 25 bp. The probability that the sequencer records the wrong nucleotide at any given base is set to 0.005. In this study we use the hg19 as reference for data simulation and analysis.

#### 2.4.2 Real RNA-seq data

The first real RNA-seq dataset includes 384 cells from a triple-negative breast cancer cell line (MDA-MB-231), half of which were treated with metformin. Two independent cell batches were captured and prepared using the Fluidigm C1 system with 96-well plate. Each batch consists of 96 treated cells and 96 untreated cells. For batch 1, two Illumina HiSeq 2500 machines are used for sequencing of the treated and control cells separately. For batch 2 the order of machines is reversed. The length of sequence reads is 100 bp. We remove samples with 0 cells; the library sizes of the rest of the samples range from 1 to 10 million.

A second real RNA-seq dataset with qPCR validated expression is obtained from the Sequencing Quality Control Consortium (SEQC) project (Su *et al.*, 2014). The dataset includes two RNA samples: sample A (universal human reference tissue, UHRR) and sample B (human brain tissue, HBRR). Replicates of these two samples were distributed to multiple laboratory sites for RNA-seq library construction and read sequencing. We select four replicates for each sample which were sequenced at the official sequencing site at Beijing Genomics Institute (BGI). The reads were sequenced using Illumina HiSeq 2000 and can be downloaded from the Sequence Read Archive (SRA). For sample A, the accession numbers of the four replicates are SRR896664, SRR896679, SRR896695 and SRR896711; for sample B they are SRR896743, SRR896759, SRR896775 and SRR896791. We also download another set of RNA-seq data sequenced by the SOLiD 5500 platform. For sample A, the accession numbers of the four replicates are SRR898783, SRR898784, SRR898790 and SRR898803; for sample B they are SRR898887, SRR898847, SRR898848 and SRR898855. The expression at gene level has been quantified independently with RT-qPCR. The validation profile contains a total of 20 801 records for each sample, which is obtained from Gene Expression Omnibus (GEO). The GEO accession numbers of sample A and sample B are GSM1361812 and GSM1361813, respectively.

### 2.5 Method comparisons

Many computational tools have been developed for isoform quantification in recent years (Zhang *et al.*, 2017). To evaluate the performance of XAEM, we select four recent advanced tools and use their latest available versions, including Salmon v0.12.0 (Patro *et al.*, 2017), Kallisto v0.45.0 (Bray *et al.*, 2016), Sailfish v0.10.0 (Srivastava *et al.*, 2016) and Cufflinks v2.2.1 (Trapnell *et al.*, 2012). Cufflinks has been widely used for isoform-level expression quantification. Salmon, Kallisto and Sailfish implement the alignment-free approach that avoids precise alignments for isoform expression quantification. The latter three methods show superior performance in both speed and accuracy (Bray *et al.*, 2016; Patro *et al.*, 2017). The commands to run each method for both indexing and quantification are presented in Supplementary Section S6. As a measure of estimation accuracy, we calculate the absolute proportion error (APE) using the estimated expression value (E) divided by the true value (T) as shown in formula (9). For simulated data, we report the median error of each isoform across 100 simulated samples as the final APE:

(9) e=|E−T|/(T+1).

## 3 Results

### 3.1 XAEM achieves higher accuracy compared with other methods in simulated data

We start the evaluation of XAEM’s performance using the simulated datasets under two scenarios of read distribution, i.e. the uniform (without bias) and non-uniform (with bias) settings. Under the uniform setting we expect the methods to perform similarly, while we would expect some differences between methods under the non-uniform setting.

#### 3.1.1 Uniform setting

To get a comprehensive comparison, the isoforms are divided into three categories according to the source genes: (i) singletons, (ii) multiple isoforms excluding paralogs (‘non-paralogs group’) and (iii) multiple isoforms which are paralogs (‘paralogs group’). ‘Singletons’ are isoforms originating from the genes with only one isoform, while ‘multiple isoforms’ are those from genes with more than one isoform. Paralogs, which have been discussed in more detail in Section 2.2.3, are isoforms with extremely similar sequences, which are difficult to distinguish from each other. The expression estimation for singletons is trivial, simply counting the fragments aligned to the gene, but the quantification for multiple isoforms is more complex due to exon-sharing. There is a total of 14 446 singletons and 31 950 multiple isoforms, where 25 838 are non-paralogs and 6112 paralogs.

Table 2A shows that the median APE of each quantification method for the uniform RNA-seq data. For the singleton group, the median error of XAEM, Salmon, Kallisto and Sailfish is 0, indicating these methods estimate the expression accurately. Cufflinks has the worst performance with an APE at 0.28. For the non-paralog group, XAEM and Salmon have a median APE at 0.18, which is slightly better than Kallisto and Sailfish (0.20). For the comparison of the paralog group, we keep the default output of each method, which means the paralogs are merged in XAEM, but remain as separate isoforms in the other methods. So this is not exactly a fair comparison, but it is given only to illustrate the problem of not considering the paralog information. Table 2A shows that XAEM has a median APE of 0.12 for paralogs, significantly better than that of all the other methods (≥0.45). The comparison shows the value of discovering the sets of paralogs and their expressions, and the difficulty of estimating the expression of the individual members of the paralogs.

**Table 2.** Comparison of median absolute proportion error (APE) between XAEM, Salmon, Kallisto, Sailfish and Cufflinks

		Multiple isoforms	
Methods	Singletons	Non-paralogs	Paralogs
(A) Uniform	(*N*=14 446)	(*N*=25 838)	(*N*=6112)
XAEM	0	0.18	0.12
Salmon	0	0.18	0.45
Kallisto	0	0.20	0.47
Sailfish	0	0.20	0.47
Cufflinks	0.28	0.36	0.54
(B) Non-uniform	(*N*=14 446)	(*N*=18 597)	(*N*=13 353)
XAEM	0	0.37	0.15
Salmon	0	0.42	0.966
Kallisto	0	0.44	0.969
Sailfish	0	0.45	0.968
Cufflinks	0.45	0.69	0.970

*Note*: The isoforms are divided into three categories: singletons, multiple isoforms with non-paralogs and paralogs. The errors are calculated under (A) uniform setting and (B) non-uniform setting.

#### 3.1.2 Non-uniform setting

We also compare the performance of XAEM and other methods under the non-uniform setting in Table 2B. Similar to the uniform setting, XAEM, Salmon, Kallisto and Sailfish achieve high accuracy for the trivial group of singletons with zero median APE. However, for the multiple isoforms, all methods produce worse results under the non-uniform setting. For the non-paralogs group, XAEM achieves the best performance (median APE = 0.37). The median APE of Salmon, Kallisto, Sailfish and Cufflinks are 0.42, 0.44, 0.45 and 0.69, respectively. For the paralogs group, the median APE of XAEM is 0.15, which is significantly lower than the other methods. From Table 2 we observe that Salmon, Kallisto and Sailfish have really similar performance; this is in agreement with the comparison results from another recent study (Zhang *et al.*, 2017). We compare the correlations between the true values and estimated values for different methods. As shown in Supplementary Table S3, XAEM estimates have a higher correlation with the true values for non-paralogs and paralogs.

We note that the number of non-paralogs (18 597) and paralogs (13 353) are different from the uniform setting. In XAEM we update the X matrix to learn the bias structure from the observed data. For different datasets, i.e. uniform and non-uniform, the biases are not the same, leading to different X matrices. Thus, the final X matrix might be different for different input datasets. When we merge the paralogs within the updated X matrix, the number of paralogs might vary between datasets. Compared to Salmon, XAEM achieves higher accuracy in 71% of the multiple isoforms (22 740 out of 31 950). Supplementary Figure S2a shows the comparison for the non-paralogs group. For the paralogs group, the out-performance of XAEM can be clearly observed in Supplementary Figure S2b. In Supplementary Figure S3, we calculate an error ratio using the APE of XAEM against Salmon for the 8933 multiple-isoform CRPs. The result also shows that XAEM has higher accuracy for the multiple isoforms. With the true values available, we find that for some paralogs, Salmon, Kallisto and Sailfish assign the total read counts from the member isoforms to only one isoform, which leads to an inferior estimation. In Supplementary Section S7, we show an example that Salmon and Kallisto allocate the total reads of paralogs NR_120496 and NR_120497 to only NR_120497, while for NR_120496 it is zero. We use the BLAST tool to compare their sequence composition (Zhang *et al.*, 2000). The alignment shows that there are 112 bp discrepancies between these two paralogs, which means they are not the same isoforms. It is unclear how Salmon and Kallisto distribute the reads to the member of a paralog set even that they have different sequences.

### 3.2 XAEM outperforms other methods in qPCR-validated data

The SEQC study (Su *et al.*, 2014) provides a unique resource of RNA-seq data with qPCR values available for independent validation. Since the validated expression values are at gene level, we first convert the expression to gene level by adding up the expression at isoform level. We calculate the correlation between the estimates and true values, and use the median absolute correlation as the measurement for each method. As shown in Table 3A and B, the correlations of XAEM, Salmon, Kallisto and Sailfish are similar (all 0.85 for Illumina and ∼0.8 for SOLiD). This is possibly due to the comparison is done at gene level, by which there is no advantage for any of these methods. In these comparisons Cufflinks performs poorly.

**Table 3.** Correlation at gene level between qPCR-validated values and estimates from different methods

	XAEM	Salmon	Kallisto	Sailfish	Cufflinks
(A) Illumina data	0.85	0.85	0.85	0.85	0.22
(B) SOLiD data	0.82	0.80	0.80	0.80	0.23
(C) Illumina versus SOLiD	0.73	0.62	0.62	0.62	0.19

*Note*: (A) The correlation is calculated using the estimates from Illumina RNA-seq data. (B) The correlation is calculated using the SOLiD data. (C) Agreement between estimates using Illumina data and SOLiD data.

We also compare the agreement between the estimates using Illumina data and SOLiD data for different methods. As shown in Table 3C, the correlation for XAEM is 0.73 at gene level, while for Salmon, Kallisto, Sailfish and Cufflinks they are 0.62, 0.62, 0.62 and 0.19, respectively. Since there are potentially different biases between Illumina and SOLiD platforms, the results indicate that the bias correction in XAEM is better than the other methods.

The SEQC project provides the qPCR primer sequence data. Some primers map uniquely to single isoforms, so these can be used to compare the methods at isoform level. We obtain the qPCR primer sequences and map them to the isoforms using Rapmap (Srivastava *et al.*, 2016). Since all methods perform similarly for singleton genes, we first select 2540 qPCR primers that map uniquely to single isoforms in multiple-isoform genes. However, unique *in-silico* mapping to an isoform is not sufficient to guarantee biologically specific binding, since this isoform may also bind to other primers. In the terminology of Section 2, it might be a neighbor of other isoforms, thus increasing the chance of non-specific binding. So, to increase specificity, we add an extra requirement that the isoform does not appear in the equivalent classes of other primers. This reduces the number of uniquely mapped isoforms to 331. For this subset, the correlations between the qPCR values and XAEM, Salmon, Sailfish, Kallisto and Cufflinks estimates are 0.84, 0.81, 0.81, 0.81 and 0.18, respectively. The results show a slight advantage of XAEM over the other methods at isoform level.

### 3.3 XAEM outperforms other methods in differential-expression analysis of real scRNA-seq data

Differential expression analysis is a common downstream analysis following isoform quantification. Here we compare the control versus treated groups in the MDA-MB-231 scRNA-seq dataset for the analysis. Since there is no gold standard to assess the power of detection of the differentially expressed (DE) isoforms, we perform the analysis in a training set and then validate the results in an independent validation set. We use two different settings: (i) a simple setting, where the training and validation sets are random splits within one batch; and (ii) a more demanding setting, where we use batch 1 data for training and batch2 data for validation.

To assess different methods, we calculate the rediscovery rate (RDR), which is the proportion of significant DE isoforms from the training set that are also significant in the validation set (Ganna *et al.*, 2014). Since the quantification accuracy of Salmon, Sailfish and Kallisto is similar (Zhang *et al.*, 2017), here we select Salmon, Cufflinks and XAEM for comparison. A counts-per-million (CPM) value is calculated to standardize the raw read count, which is then log scaled and normalized by the median CPM. The expression levels between the control and treated groups are compared using t-test with a threshold of *P*-value <0.05. For each method, we take the top *M* statistically significant DE isoforms observed from the training set to the validation set and calculate the RDR. In Supplementary Figure S4, we first show that the RDRs of all the methods are unbiased by performing the DE analysis under the null hypothesis. A completely inaccurate quantification method will generate mostly noise and have a low RDR ≈ 0.05; thus a higher RDR indicates better quantification (Ganna *et al.*, 2015).

**Fig. 3.**
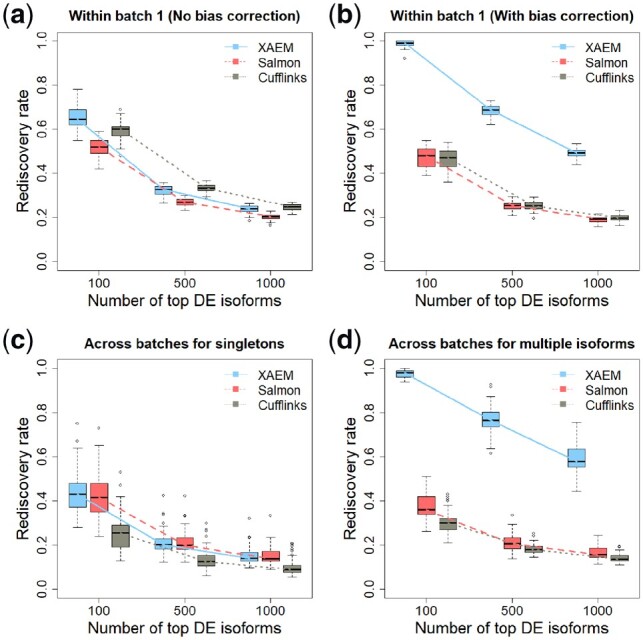
Detection and validation of differentially expressed (DE) isoforms using the MDA-MB-231 scRNA-seq dataset. XAEM, Salmon and Cufflinks are presented in blue-solid, red-dashed and grey-dotted lines, respectively. The x-axis shows the number of top DE isoforms in the training set; the y-axis is the proportion of rediscovery in the validation set. The rediscovery rate (RDR) is calculated by comparing the top 100, 500 and 1000 DE isoforms from the training set with all the significant DE isoforms from the validation set. The boxplots show the RDR from 50 times’ run. (**a**) Both training set and validation set are constructed using cells from batch 1. The quantification of XAEM, Salmon and Cufflinks is performed without bias correction. (**b**) The quantification from the three methods are bias-corrected. (**c**) The training set is constructed using cells from batch 1, while the validation set uses cells from batch 2. The RDR is calculated for only singleton isoforms. (**d**) The training set is constructed using cells from batch 1, and the validation set using cells from batch 2. The RDR is calculated using only non-paralogs. (Color version of this figure is available at *Bioinformatics* online.)

We first investigate results of DE analysis within the same batch, thus removing potential batch-related effects. This is done for both batch 1 and 2 separately. Here we show the results using batch 1, which contains 96 treated and 96 untreated cells. A total of 40 treated and 40 control samples are randomly selected from batch 1 to generate the training set, then *a different set of 40 treated and 40 control samples are selected for the validation set.* To avoid the effect of randomness in the selection of training and validation sets, the whole analysis procedure is repeated 50 times. The RDR is calculated by comparing the top 100, 500 and 1000 DE isoforms from the training set with all the significant DE isoforms from the validation set. We exclude the paralogs in the comparisons since Salmon and Cufflinks can have poor estimates; the result for paralogs is shown in Supplementary Figure S5. Figure 3a and b show the results using the samples from batch 1. In Figure 3a all methods are run without bias correction. For XAEM, this means that the *X* matrix is not updated. Figure 3a shows that the methods perform similarly without bias correction, and the performance of Salmon is even slightly worse than Cufflinks.

In Figure 3b, the bias correction step is applied to all methods. For XAEM it means that the full AEM algorithm is used to estimate both the design matrix *X* and *β*. Figure 3b shows a notable improvement of the RDR in XAEM. For the top 100 DE isoforms, the RDRs for XAEM, Salmon and Cufflinks are 1.0, 0.56 and 0.50, respectively. Almost all the 100 DE isoforms from the training set are re-discovered in the validation set by XAEM, while only 56 are re-discovered using Salmon and 50 using Cufflinks. The same pattern can be observed from the results for batch 2 (Supplementary Fig. S6). For the top 500 DE isoforms, the RDR of XAEM is 0.73, which is higher than Salmon at 0.28 and Cufflinks at 0.26. The RDR decreases along the x-axis, which means that when the number of top DE isoforms increases, a smaller proportion of them can be validated from the training set to the validation set. Overall, the bias correction works well for XAEM. The results in Figure 3b indicate that the bias correction by updating *X* matrix using the AEM algorithm is the key feature that makes XAEM achieve better performance.

We further compare the performance across batches, which is harder to achieve. In the MDA-MB-231 dataset, the control and treated samples are separately sequenced in two different machines. In batch 1, the control samples are sequenced in machine 1, and the treated samples in machine 2. However, in batch 2, the machines are reversed: the control samples are sequenced in machine 2 and the treated samples in machine 1. Thus, there might be a machine effect between the two batches. We estimate the machine effect and remove it ahead of the differential-expression analysis. The details of this adjustment is illustrated in Supplementary Section S8. In this comparison we split the isoforms into two groups: (i) singletons, in which we do not expect a difference between methods, and (ii) multiple isoforms, which are non-paralogs. For each method, we calculate the RDR based on the top 100 to 1000 significant DE isoforms in the training set. Figure 3c shows the result for the singletons. As expected, the RDRs of XAEM and Salmon are similar. This is in agreement with what we observe from Table 2, where the quantification of singletons is the same for these two methods. However, for multiple isoforms, Figure 3d shows that XAEM achieves a much higher RDR than Salmon and Cufflinks. For the top 100 DE isoforms, the RDR for XAEM, Salmon and Cufflinks is 0.97, 0.38 and 0.32, respectively. The overall RDR across the top 100, 500 and 1000 DE isoforms of XAEM (0.77) is about three-fold higher than that in Salmon (0.26) and Cufflinks (0.22).

## 4 Discussion and conclusion

We have developed a novel method called XAEM to estimate isoform expression from multiple RNA-seq samples. Compared to existing methods, which are implicitly based on a linear model *Xβ* with known *X*, XAEM is based on a more flexible and richer bilinear model that treats both *X* and *β* as unknown parameters. Instead of adding a correction step of presumed known biases, as is commonly done in existing methods, XAEM automatically corrects for potentially unknown biases using an AEM approach. The AEM makes use of the variation of expression from the multiple samples. XAEM also utilizes the state-of-the-art quasi-mapping to speed up the overall quantification of isoform expression. We have reported a test of computational resource usage of XAEM in Supplementary Section S9, which shows that XAEM is fast and memory efficient. Overall, we show that better statistical modelling helps XAEM achieve significantly better results in comparison with recent advanced methods in both simulated and real RNA-seq datasets.

The key difference between XAEM and existing methods such as Sailfish is in the explicit formulation of the design matrix *X*. Technically, as done in existing methods, it is possible to perform the EM algorithm without knowing *X* explicitly. However, with such an approach, it is not obvious how to generalize the linear model into a much richer bilinear model as we have described. The existing methods also use a transcriptome-wide *X*, which would be too high-dimensional to treat as an unknown parameter. XAEM uses the concept of transcription clusters, thus breaking the transcriptome into smaller units; this limits the number of *X*-parameters substantially, and makes the procedure computationally and statistically feasible. The initial *X* matrix in XAEM is generated using uniform assumption. We have also investigated the effects using the biased assumptions. As shown in Supplementary Section S10, the results are similar to those based on the uniform assumption. We have used the VBEM algorithm inside XAEM, but in principle any version of EM algorithm can be used, e.g. the alternative squared-EM (Varadhan and Roland, 2008). Using other EM algorithms within XAEM is worth further investigations.

The comparison in Table 2 shows that XAEM achieves a better performance for the multiple isoforms. In this analysis we used the same simulation tool Polyester as we used to construct the X matrix. There might be some potential effects on the comparison analysis. However, as expected in the uniform case, XAEM performs similarly with Salmon for singletons and non-paralogs. This shows that there is no special advantage for XAEM in the simulation setup. In the comparison of the agreement between Illumina data and SOLiD data, XAEM achieves a higher correlation than the other methods. Given the potential differences of the biases between the two platforms, the result shows that XAEM has a better performance in the bias correction of real RNA-seq data.

From the comparison we also see the difficulty to quantify the expression of individual paralogs. It is not obvious how paralogs should be used in pathway enrichment analysis; this merits further studies. As XAEM reports the complete set of paralogs, users can try out several methods. For example, suppose there is a merged paralog involving three genes: A, B and C. If there is a pathway involving any of these genes, say gene C, then we might consider using the total paralog-level expression for this gene, or the average expression (total/3). Without the paralog information, we do not have this option, and may unknowingly use inappropriate expression value. The merging of paralogs in XAEM at least makes the quantification process transparent.

In principle XAEM can be used for any bilinear model *μ* = *Xβ*, which, for example, looks appropriate to the data from tiling microarrays. As before, *β* represents the underlying transcript intensity; *μ* represents the probe intensities; the *X* matrix represents the mapping of the set of transcripts that theoretically can contribute to each probe. The starting *X* matrix must be derived from the assumed gene model in the transcriptome annotation file rather than learned computationally as we have described. One weakness is that the gene model may not tell us much about paralogs, which will likely lead to a lot of non-specific binding. Mueckstein *et al.* present an improved thermodynamic model for microarray hybridization, which may allow assessment of non-specific binding due to the cross-hybridization among paralogs (Mueckstein *et al.*, 2010). Showing how this works in practice will be worth further investigations.

Performances of XAEM depend on multiple samples for updating the design matrix *X*, so it is desirable to make XAEM more robust against sample heterogeneity. We can do this by extending the model to allow multiple *X* matrices within each gene (CRP); for example, we can start with a clustering step to the samples, and assign one *X* for each cluster. Theoretically, distinct *X* matrices should produce distinct vector spaces, and these are likely learnable from the observed count data. Developing such an extension is a worthy project, but beyond the scope of the current paper. For now, in practice we recommend the use of the algorithm in batches of reasonably homogenous samples such as the same tissue or cancer type.

In summary, we have introduced XAEM, an efficient and accurate method to estimate isoform expression from multiple RNA-seq samples. Compared with other competing methods, XAEM achieves better performance in estimation accuracy and rediscovery rate of DE isoform from RNA-seq data.

## Funding

This work is partially supported by funding from the Swedish Cancer Fonden, the Swedish Research Council (VR), the Swedish Foundation for Strategic Research (SSF) and China Scholarship Council (CSC).

*Conflict of Interest*: none declared.

## Supplementary Material

btz640_Supplementary_Data
