# Supplementary material for: Alternating EM algorithm for a bilinear model in isoform quantification from RNA-seq data
Source: Bioinformatics. 2019 Aug 10;36(3):805–12. doi: 10.1093/bioinformatics/btz640 (PMC9883676; doi:10.1093/bioinformatics/btz640)
Supplement: btz640_Supplementary_Data [file bioinformatics_36_3_805_s4.docx]

**Alternating EM algorithm for a bilinear model in isoform quantification from RNA-seq data**

**Wenjiang Deng *et al*.**

Section 1: figures and tables

**Supplementary Figure S1**

**
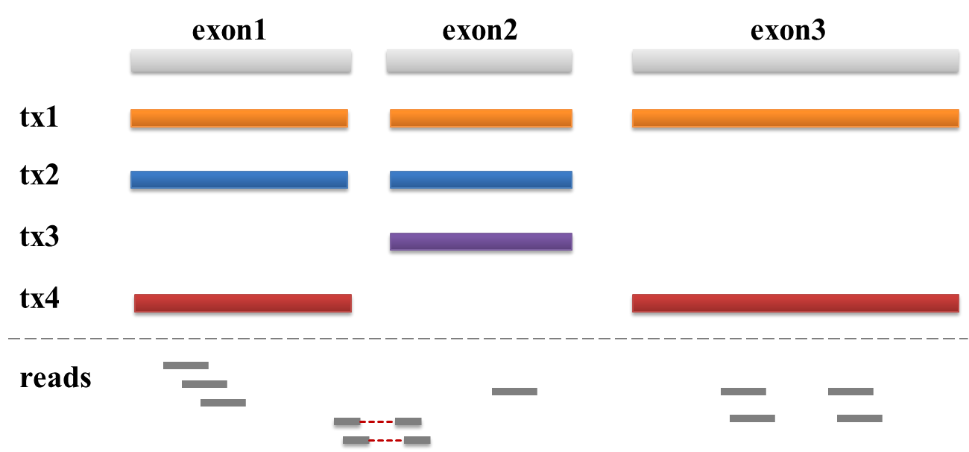
**

**Figure S1: A toy illustration of exon-sharing**. tx1, tx2, tx3 and tx4 are transcripts from the same gene consisting of three exons in total. There are three reads mapped to exon1, which is shared by three transcripts tx1, tx2 and tx4. This makes it impossible to assign these reads to any specific transcript. For simplicity, single-end reads and exons are presented in the figure. The exon-junction spanning reads are also shown. In practice the exon-sharing concept applies in a more abstract form in terms of paired-end reads and equivalence-classes.

**Supplementary Figure S2**


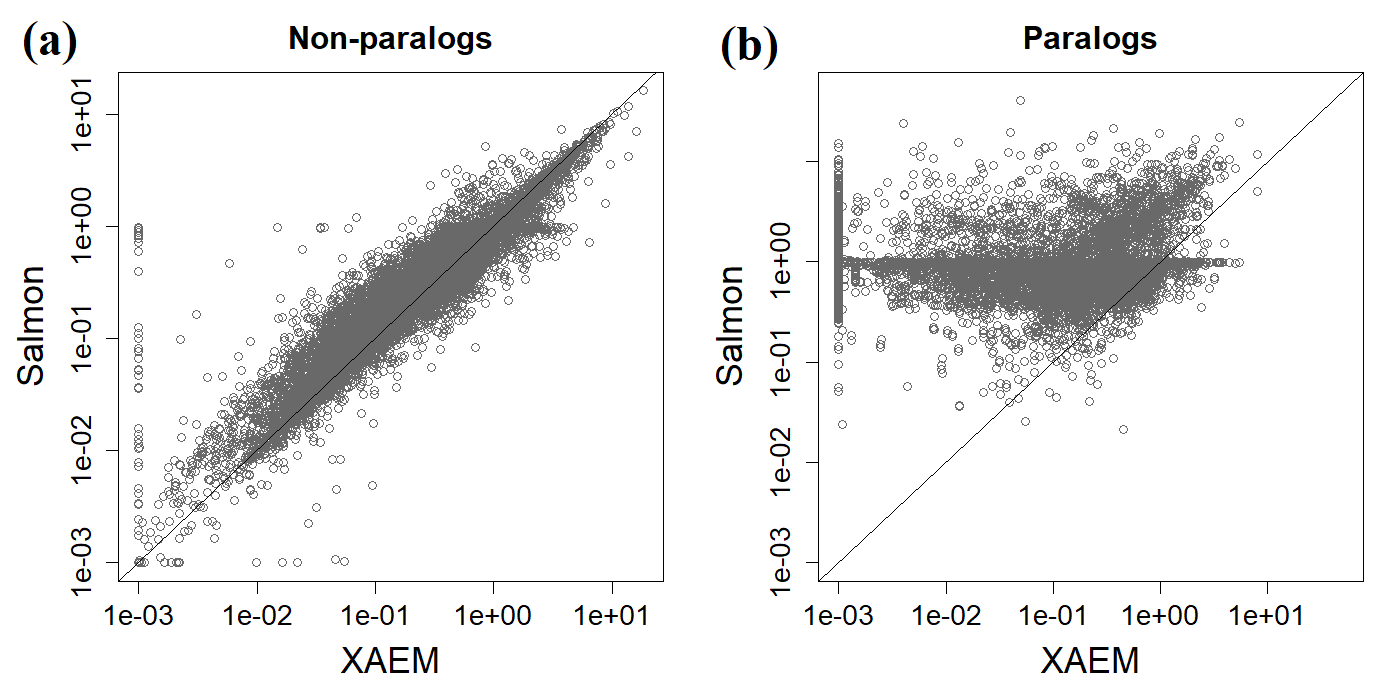


**Figure S2: Comparison of estimation errors between XAEM and Salmon under non-uniform setting.** (a) Multiple isoforms with non-paralogs. (b) Multiple isoforms with paralogs. APEs are in log scale with a small value (0.001) added to avoid log-zero. The solid line is the diagonal line

**Supplementary Figure S3**


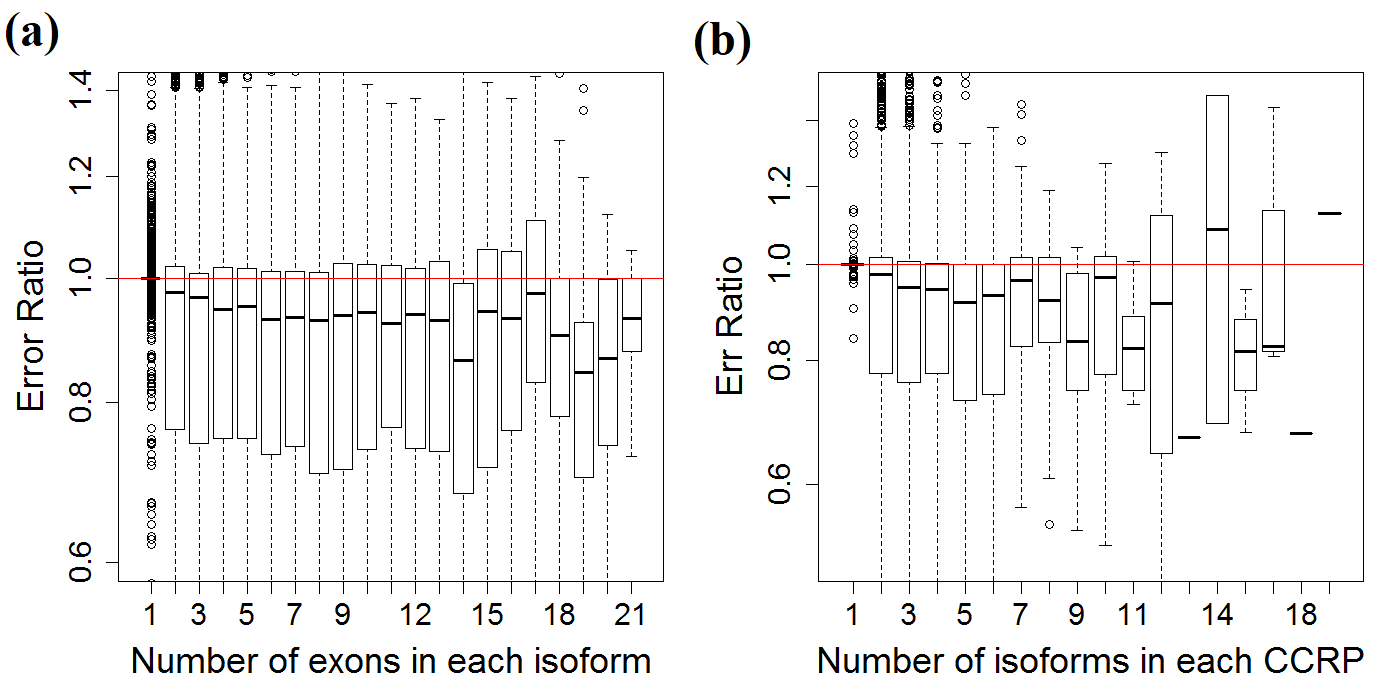


**Figure S3:** Comparison of error ratio between XAEM and Salmon. An error ratio is calculated using the APE of XAEM dividing the APE of Salmon for the multiple isoforms from 8,933 CCRPs. (a) The boxplot is drawn based on the number of exons in each isoform. (b) The comparison is based on the number of isoforms in each CCRP.

**
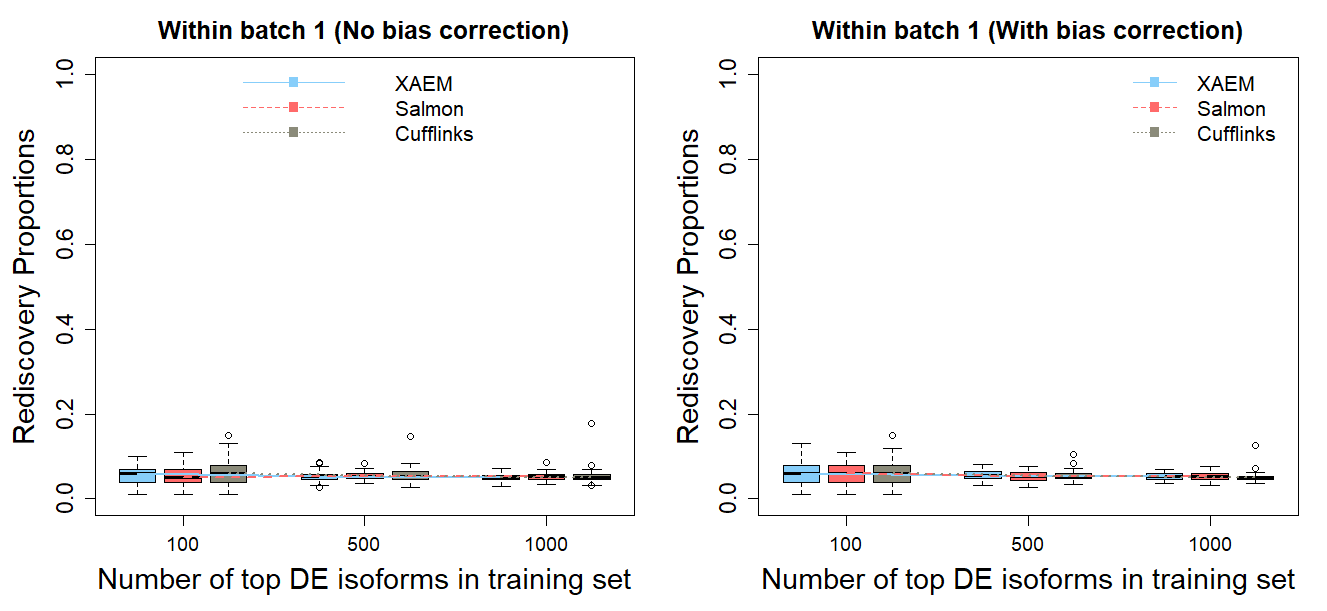
Supplementary Figure S4**

**(b)**

**(a)**


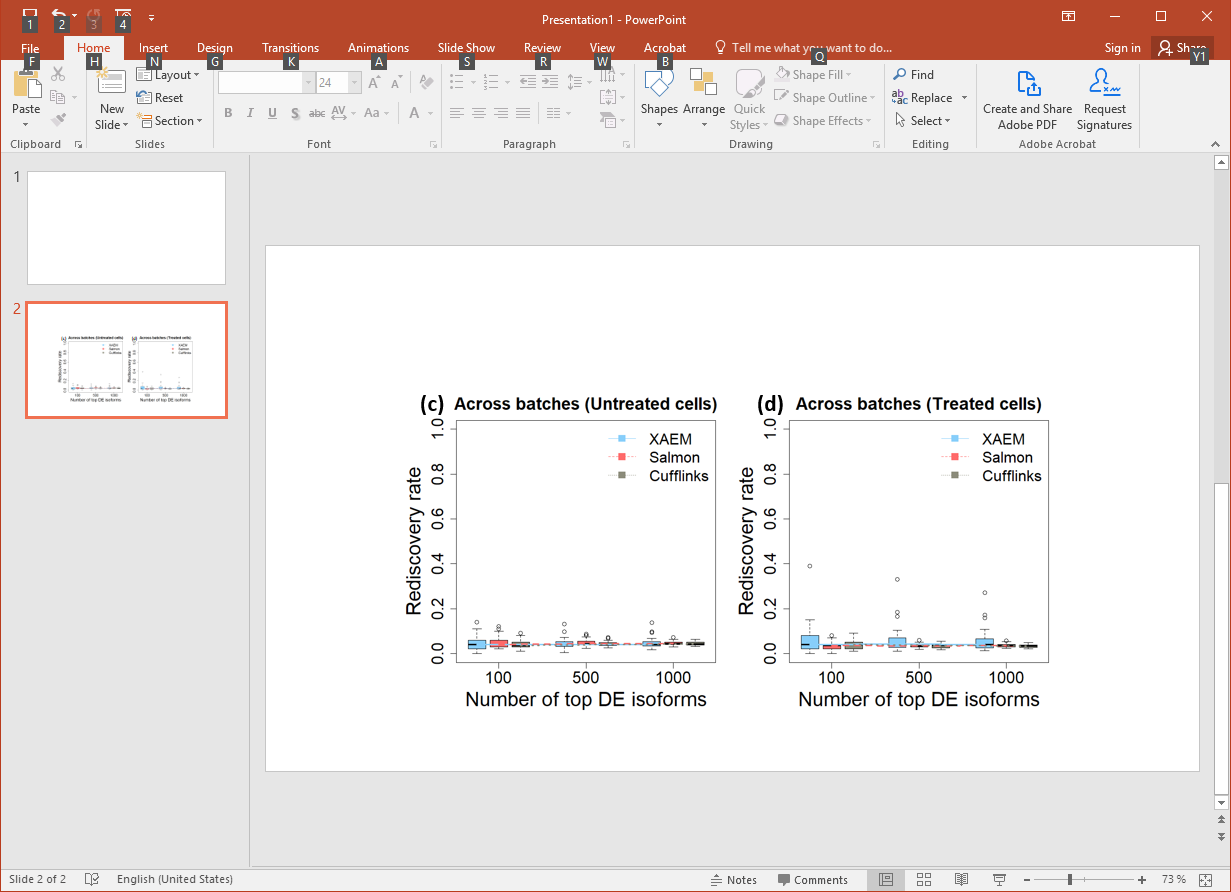


**Figure S4: Differential-expression (DE) analysis under null hypothesis:** **the plots indicate that the RDR metric for each of the methods is unbiased.** The comparison is made groups *within* the untreated or within the treated groups, so we do not expect any differentially expected isoforms (hence null hypothesis) and the RDRs should be close to its target value of 0.05. For (a) and (b), the training and validation sets are constructed using only untreated samples from Batch 1. The training set contains randomly selected 20 vs 20 cells, and validation set contains separate 20 vs 20 cells. This comparison is repeated 50 times; the RDRs are summarized in boxplots. (a) The isoform estimations from XAEM, Salmon and Cufflinks are not bias-corrected; (b) The same as panel (a) for bias-corrected estimates. For (c) and (d), the training set contains randomly selected 40 vs 40 cells from Batch 1, and the validation set 40 vs 40 cells from Batch 2. (c) The same as panel (b) but across batches; (d) The same as panel (d) for treated cells.

**Supplementary Figure S5**

**
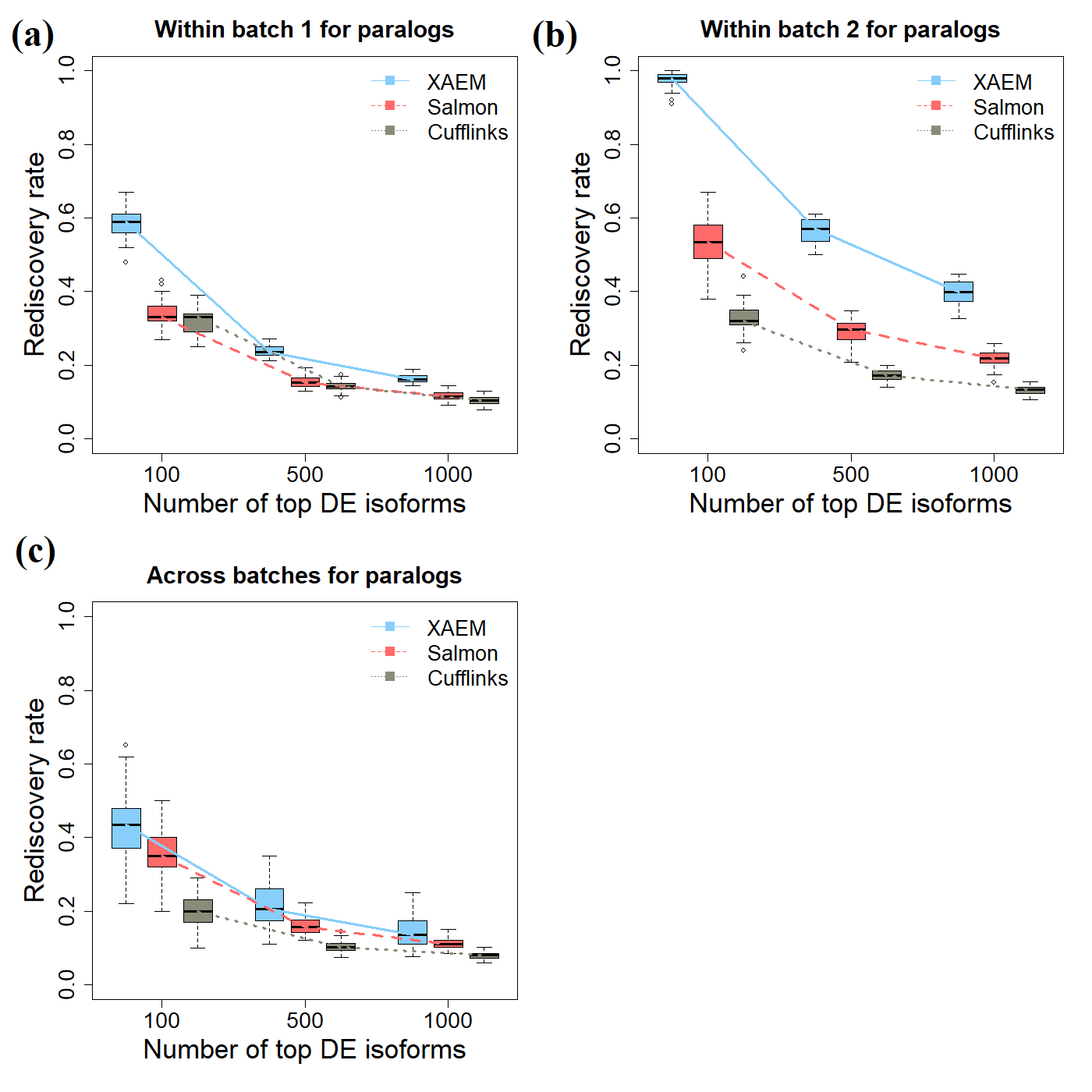
**

**Figure S5: Detection and validation of DE isoforms for paralogs.** (a) Both training set and validation set are constructed using samples from batch 1. The isoform estimations from XAEM, Salmon and Cufflinks are bias-corrected. (b) Both training set and validation set are constructed using samples from batch 2. (c) The training set is constructed using samples from batch 1 and validation set from batch 2.


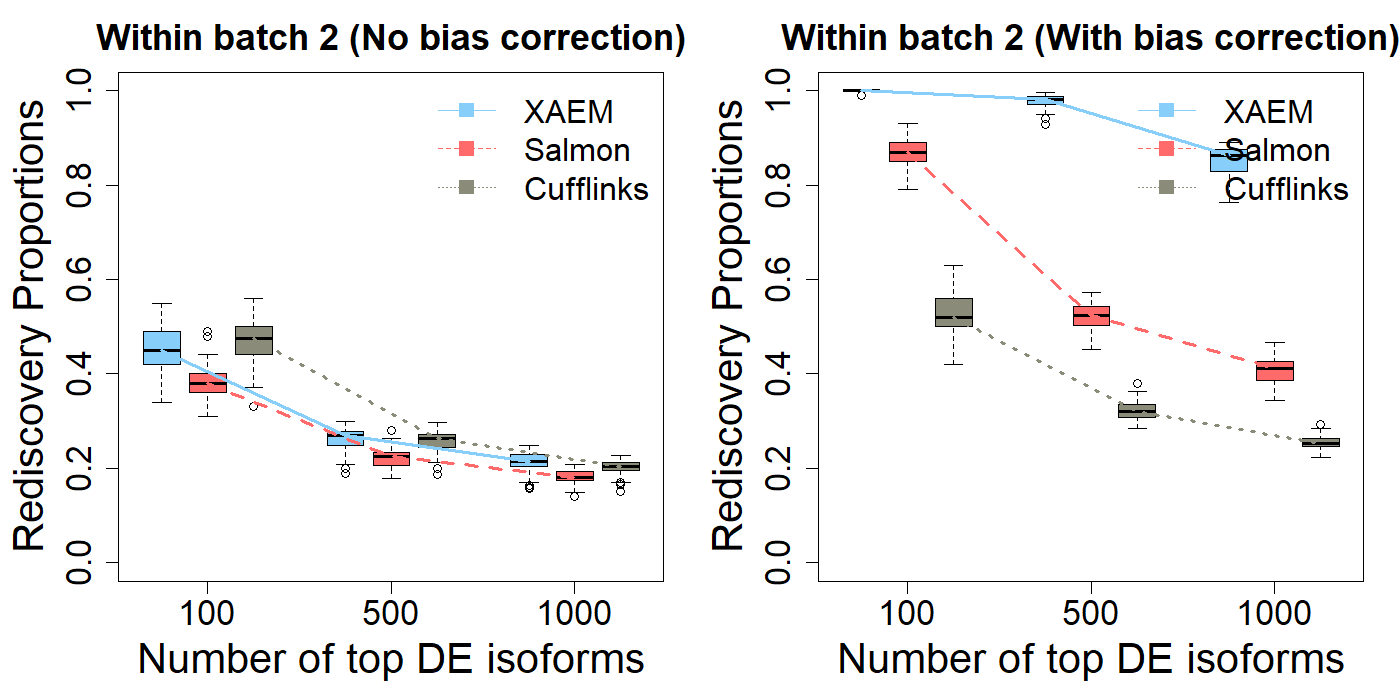
**Supplementary Figure S6**

**Figure S6: Detection and validation of DE isoforms using the samples from batch 2.** (a) The isoform estimations from XAEM, Salmon and Cufflinks are not bias corrected. (b) The estimates from three methods are bias-corrected.

**Supplementary Table S1**

**Table S1**. Comparison of absolute proportional error (APE) between no filter (H=0) setting and H=2.5% setting. The non-uniform simulated data were used for quantification and the errors were calculated in non-paralog group (*N*=20629).

|  | Min. | 1st Qu. | Median | Mean | 3rd Qu. | Max. |
| --- | --- | --- | --- | --- | --- | --- |
| No filter | 0 | 0.09747 | 0.3063 | 0.5033 | 0.7686 | 16.05 |
| H=2.5% | 0 | 0.09796 | 0.3063 | 0.5033 | 0.7681 | 15.91 |

**Supplementary Table S2**

**Table S2**: Comparison of median APE between XAEM and Salmon for multiple isoforms. The original expression profile of the HCT116 cell line is used. Isoforms from each X matrix with at least one isoform expressed in ≥75% samples are kept for comparison.

|  | Non-paralogs  (*N*=474) | Paralogs  (*N*=2,133) |
| --- | --- | --- |
| XAEM | 0.40 | 0.01 |
| Salmon | 0.37 | 0.57 |

**Supplementary Table S3**

**Table S3**. Comparison of correlation between the true read counts and estimated values from each method. The correlation is calculated for uniform and non-uniform simulated data, respectively.

| Methods | Singletons | Non-paralogs | Paralogs |
| --- | --- | --- | --- |
| 1. Uniform | **(N=14446)** | **(N=25838)** | **(N=6112)** |
| XAEM | **1** | **0.89** | **0.95** |
| Salmon | **1** | **0.83** | **0.63** |
| Kallisto | **1** | **0.83** | **0.65** |
| Sailfish | **1** | **0.83** | **0.66** |
| Cufflinks | **0.31** | **0.79** | **0.48** |
| 1. Non-uniform | **(N=14446)** | **(N=18597)** | **(N=13353)** |
| XAEM | **1** | **0.60** | **0.69** |
| Salmon | **1** | **0.55** | **0.23** |
| Kallisto | **1** | **0.56** | **0.25** |
| Sailfish | **1** | **0.55** | **0.25** |
| Cufflinks | **0.53** | **0.52** | **0.21** |

Section 2: An investigation of the threshold of singular value

Statistically, because the estimation of β is an inverse problem, the negative impact of tiny singular values is better seen from the pattern of their inverse. The following Figure S7 shows the pattern of the inverse of 31,631 singular values collected from 8933 multi-isoform CRPs. The ‘elbow’ of the curve marks a point where the impact of a tiny singular value becomes substantial. From the figure, this point corresponds to singular value around 0.02 to 0.03, close to the threshold we chose. The pattern does not depend of the number of samples, but is determined by the level of sequence similarity (paralogs) between the transcripts in the transcriptome annotation.


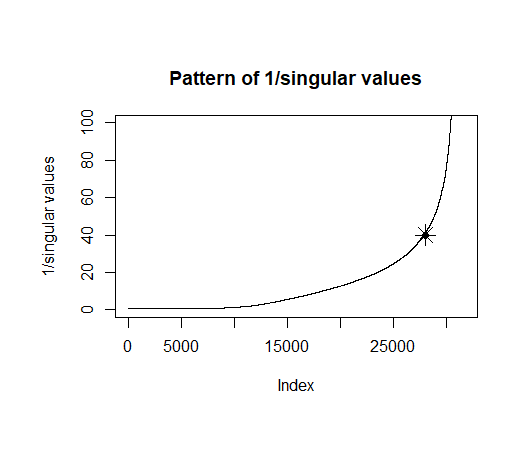


**Figure S7:** The pattern of the inversed singular values calculated from multi-isoform CRPs; the star position corresponds to singular value of 0.025.

Section 3: Merge paralogs using the k-means clustering

For each X matrix we first compute the singular values v’s in the singular value decomposition (SVD) of X. Two exact paralogs will produce v=0. Theoretically k should be the number of positive singular values, but in practice tiny singular values will still produce estimation problems because of near singularity. Here we show an example of X matrix and different thresholds to merge paralogs. The original X matrix is:

crp1

## NM_001286379 NM_001286380 NM_001286381 NM_032448
## 1000 0.06915871 0.0000000000 0.000000000 0.000000e+00
## 1101 0.37463262 0.3986940657 0.000000000 3.914341e-01
## 1111 0.55620867 0.5765315921 0.961951519 5.846968e-01
## 0100 0.00000000 0.0244862685 0.000000000 0.000000e+00
## 0101 0.00000000 0.0002880737 0.000000000 9.624639e-05
## 0010 0.00000000 0.0000000000 0.029456889 0.000000e+00
## 0011 0.00000000 0.0000000000 0.008591593 0.000000e+00
## 0001 0.00000000 0.0000000000 0.000000000 2.377286e-02

newx = crp1
sval = svd(newx)$d; ## **singular values**con = max(sval)/sval ## **condition numbers to determine which transcripts will be merged**

con

## [1] 1.000000 3.340534 24.999660 61.054808

## condition numbers of the four colunms

crp2=ccrpfun(crp1)

## ccrpfun() is the function to merge paralogs. The default threshold in ccrpfun() for the condition number **is 50 (singularvalue 0.02)**. In crp1, the column with condition number of 61, i.e. ‘NM_032448’, will be merged in crp2 as shown below.

crp2

## NM_001286379 **NM_001286380+NM_032448** NM_001286381
## 1000 0.06915871 0.0000000000 0.000000000
## 1101 0.37463262 0.3950640685 0.000000000
## 1111 0.55620867 0.5806142080 0.961951519
## 0100 0.00000000 0.0122431342 0.000000000
## 0101 0.00000000 0.0001921601 0.000000000
## 0010 0.00000000 0.0000000000 0.029456889
## 0011 0.00000000 0.0000000000 0.008591593
## 0001 0.00000000 0.0118864293 0.000000000

## ‘NM_032448’ and ‘NM_001286379’ are identified as paralogs and merged together in crp2

newx=crp2
sval = svd(newx)$d; ## singular values
con = max(sval)/sval ## condition numbers
con

## [1] 1.000000 3.245175 25.449144 ## condition numbers of crp2

newx=crp2
crp3=ccrpfun(crp2,clim=20)

crp3

## **NM_001286379+NM_001286380+NM_032448** NM_001286381
## 1000 3.457935e-02 0.000000000
## 1101 3.848483e-01 0.000000000
## 1111 5.684114e-01 0.961951519
## 0100 6.121567e-03 0.000000000
## 0101 9.608003e-05 0.000000000
## 0010 0.000000e+00 0.029456889
## 0011 0.000000e+00 0.008591593
## 0001 5.943215e-03 0.000000000

## If we choose a threshold at 20 then ‘NM_001286379’ will also be merged with ‘NM_001286380 NM_032448’. However, from crp2 we can see that ‘NM_001286379’ is not that close to ‘NM_001286380 NM_032448’, which means ‘NM_001286379’ is a false positive paralog due to the small threshold that we use.

Section 4: A test of XAEM using different tissue data from GTEx project

**
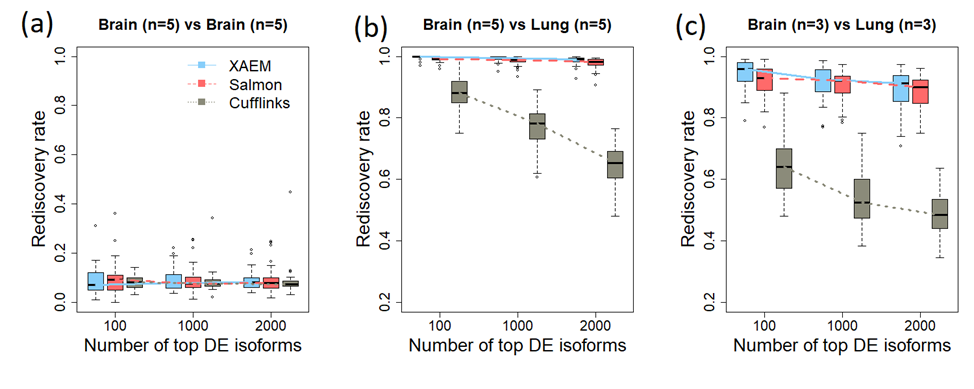
Figure S8:** Differential-expression (DE) analysis using the GTEx data. Each boxplot summarizes the rediscovery rates from 50 independent validation runs. (a) Under null hypothesis, using 5 random samples from brain vs another set of 5 samples also from brain. (b) using 5 random samples from brain vs 5 random samples from lung, and (c) using 3 random samples from brain vs 3 random samples from lung.

To test XAEM on different tissues, we downloaded the RNA-seq data for 20 brain and 20 lung samples from the GTEx project. We then compare the performance of XAEM with Salmon and Cufflinks in a differential-expression (DE) analysis. The rediscovery rate (RDR) statistics are as given in the paper; to account for sampling variability, the comparisons are based on 50 independent validation runs.

We first show all the methods are unbiased by performing DE analyses under the null hypothesis; for this we compare 5 random samples from brain vs another set of 5 samples also from brain. Figure S8(a) shows that the rediscovery rate for all three methods is near the target value 0.05. In Figure S8(b), we compare 5 random samples from brain vs 5 random samples from lung. It can be seen that XAEM and Salmon have much higher RDRs than Cufflinks. However, because the tissues are so different, even with n=5 the statistical power is too large, resulting in RDRs being too close to 1 for XAEM vs Salmon, so it is difficult to compare them. So we decrease the number of samples to 3 vs 3 in Figure S8(c). The result shows XAEM gets slightly higher RDR than Salmon. This result indicates that optimizing the design matrix is not an issue for data from different tissues.

Section 5: A test of XAEM using heterogeneous tissue data from GTEx project


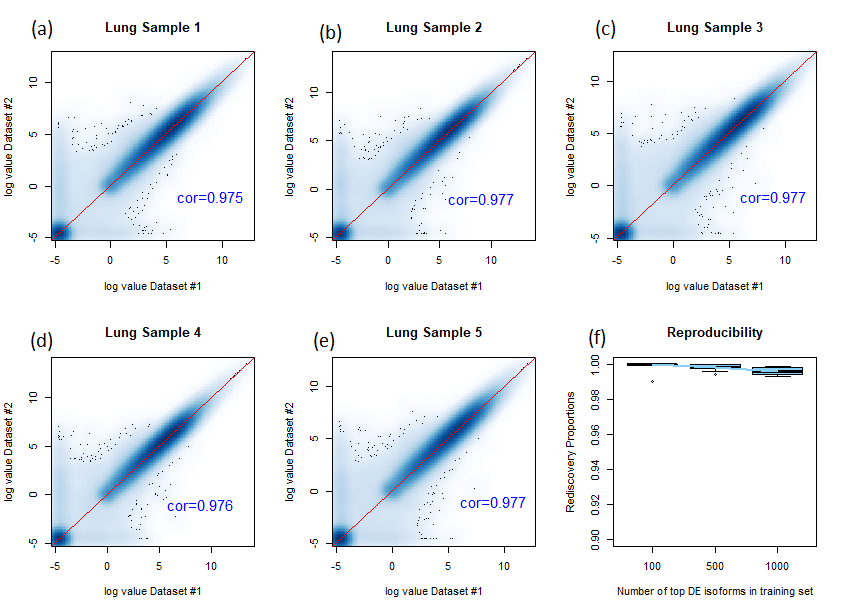
To investigate the performance of XAEM with heterogeneous input samples, we performed a study described as follows. We downloaded 50 lung samples data from GTEx to create Dataset #1; for Dataset #2 it consists of 45 samples from 9 tissues that together with 5 lung samples from Dataset #1. The 9 tissues are: muscle, skin, heart, breast, stomach, testis, liver, spleen and prostate. We separately ran XAEM for each dataset, and report the correlations of the 5 shared lung samples between two datasets in Figure 9 (a-e). The correlations across 5 lung samples are ~0.98, indicating that the estimations of these common samples between two datasets are highly consistent. We also calculated the absolute proportion error (APE) between the two runs; the median APE across the 5 samples is 0.004.

**Figure S9:** Panels of a-e: scatter plots of log-expression of the 5 overlapping lung samples from Dataset #1 and Dataset #2. (f) Reproducibility of DE analyses between the two datasets; note y-axis from 0.9 to 1.

We further investigated the reproducibility of 5 shared lung samples between the two datasets by differential-expression (DE) analysis. We first performed DE analyses between 5 shared lung samples vs 5 samples from each of the other 9 tissues. In each case we identified the top 100, 500 and 1000 isoforms. We then performed similar analysis but using the expression of the 5 shared lung samples from Dataset #1. The box plots in Figure 9(f) summarize the re-discovery rate for those top 100, 500 and 1000 isoforms (note the y-axis from 0.9 to 1). The re-discovery rates are all close to 1, indicating that the DE analyses are highly reproducible between Dataset #1 and Dataset #2. Overall the results show that the performance of XAEM is robust with respect to the heterogeneity of samples.

Section 6: Commands to run different methods for isoform quantification

In this study we mainly use the default options to run each method according to their manuals. Specifically, the commands for indexing the reference transcriptome and quantifying the isoform expression are as follows:

**Kallisto:**

kallisto index -i kaindex reference.fa

kallisto quant -i kaindex --bias --plaintext fq1 fq2 -t 8

**Salmon:**

salmon index -t reference.fa -i salmonindex

salmon quant -i salmonindex --seqBias --gcBias --posBias --biasSpeedSamp 5 -l IU -1 fq1 -2 fq2 -p 8

**Sailfish:**

sailfish index -t reference.fa -o sailfishindex

sailfish quant -i sailfishindex -l IU -1 fq1 -2 fq2 --biasCorrect -p 8

**Cufflinks:**

hisat2-build reference.fa tx_index

hisat2 -f -x index -1 fq1 -2 fq2 -p 8 --summary-file outdir/summary.txt | samtools view -bS - | samtools sort - -o outdir/sample"sorted.bam"

cufflinks -o outdir -p 8 –G genes_filtered.gtf -b genome.fa outdir/sample"sorted.bam"

Section 7: An example of BLAST alignment between isoform NR_120496 and NR_120497

From the simulated data where the true expression values are available, we notice that for some paralogs Salmon and Kallisto just allocate the whole read counts to only one transcript. For example, the true read counts for paralogs NR_120496 and NR_120497 are 59 and 340 in a simulated sample. Both Salmon and Kallisto assign the total counts of these two transcripts, i.e. 399, to only NR_120497, while the expression of NR_120496 is zero. We use the BLAST (https://blast.ncbi.nlm.nih.gov/) to compare their sequence composition. Figure S10 shows that the length of NR_120496 is 3709 bp and NR_120497 3597 bp. It can be seen that there is a gap in the beginning of NR_120496. Figure S11 shows the details of the alignment in base pairs. It is easy to calculate that there are actually 112 bp discrepancies between these two isoforms, which indicates that they are not exactly the same isoforms. It is unclear how Salmon and Kallisto distribute reads to the member of a paralog set even that they have different sequences.


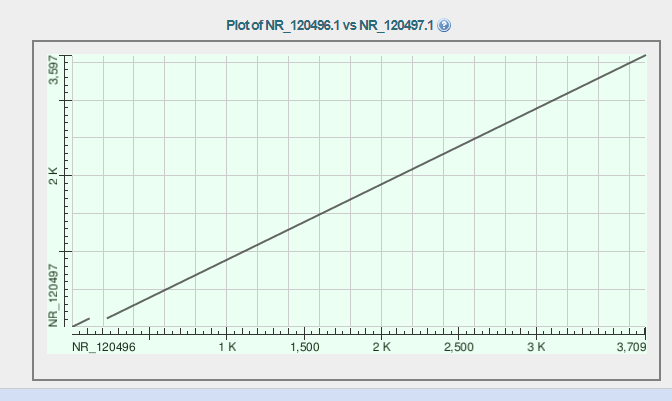


**Figure S10:** The overview of BLAST alignment between NR_120496 and NR_120497


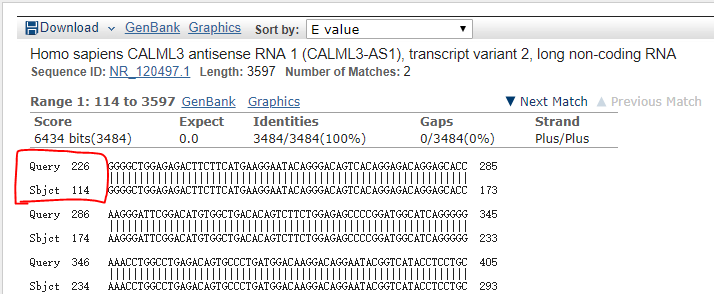

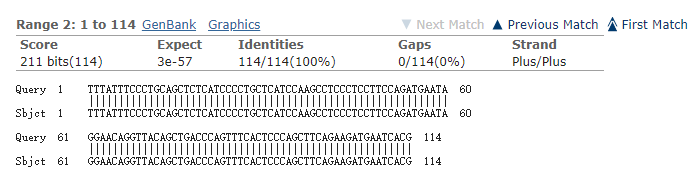


**Figure S11:** The detailed alignment in base pairs between NR_120496 and NR_120497

Section 8: Removal of confounding machine effects in differential expression analysis

In this study we use a single cell RNA-seq dataset to compare the estimation performance between XAEM with other competing approaches. The dataset contains 384 cells from a triple-negative breast cancer cell line (MDA-MB-231), half of which were treated with metformin. As shown in Table S7, two independent cell batches were captured and prepared using the Fluidigm C1 system on a 96-well format. Each batch includes 96 treated and untreated cells, respectively.

**Table S7:** the detailed information of control and treated cells within each batch

| **Batch 1** | Control 1  (96 cells) | Treated 1  (96 cells) |
| --- | --- | --- |
| **Batch 2** | Treated 2  (96 cells) | Control 2  (96 cells) |

We notice that two sequencing machines were used for control and treated cells within a batch, while the order of machines for control and treated group is reversed between batch 1 and batch 2 (Table S8). In batch 1, the control group is sequenced using machine 1 and the treated group is sequenced using machine 2. However, in batch 2, the treated group is sequenced with machine 1 and control group with machine 2.

**Table S8:** Effects between control and treated groups within each batch

|  | **Machine 1** | **Machine 2** |
| --- | --- | --- |
| **Batch 1** | Control 1  (A+M1) | Treated 1  (B+M2) |
| **Batch 2** | Treated 2  (B+M1) | Control 2  (A+M2) |

This may cause a potential problem when we do the differential expression analysis between control and treated groups because the machine effect can have confounding effect on the DE isoforms. To remove the machine effects between batches, we first assume the effect for machine 1 and 2 as M1 and M2, respectively. The effects between control and treated cells are denoted as A and B. We then use the effects of Treated 1 and Control 2 to subtract those from Control 1 and Treated 2, which is:

$\left( B+M2+A+M2 \right)-\left( A+M1+B+M1 \right)=2*(M2-M1)$.

We denote$\triangle=M2-M1$. For Treated 1 group, we adjust the machine effect using:

$B+M2-\triangle=B+M1$.

Similarly for Control 2 it becomes$A+M2-\triangle=A+M1$. The Control 1 and Treated 2 group remain the same and now each group only contains the same effect from machine 1. We then continue to do the DE analysis using the adjusted data from different batches.

Section 9: Computational resource usage of XAEM

XAEM implements the state-of-the-art quasi-mapping to speed up the read alignment step. For the AEM step XAEM utilizes parallel running for quantification of isoform expression.

We re-run XAEM for the toy dataset (http://fafner.meb.ki.se/biostatwiki/xaem/) on a high-performance computing (HPC) server (https://www.uppmax.uu.se/) using 8 cores (8 Gb per core). It took only 45 seconds for reads mapping and construction of the count matrix.

We also tested XAEM to analyze the real data from the GTEx project (https://gtexportal.org/home/). We downloaded 40 samples of brain and lung tissues, with an average of 23.6 million reads per sample. We used 8 cores on the same HPC server. The read alignment step took 1 hour and 54 minutes, which was similar to Salmon. The full AEM algorithm took 1 hour and 25 minutes, and the max memory usage was 16 GB. Overall the AEM step took 2-3 minutes per sample with a moderate memory usage.

Section 10: A test of effects when generating the initial X using biased assumptions

To test the effects of different assumptions when generating the starting X, we use two types of positional biases, rnaf and cdnaf, to re-construct the initial X. As shown in Figure S12, the rnaf bias generates more fragments in the middle of a transcript than the two ends; the cdnaf bias will cause a much higher coverage towards the 3’ end than the 5’ end. Here we denote the new X matrix as “biased X matrix” and the standard X as “uniform X matrix”. We also set the sequencing error rate at 0.01. We then apply the biased X to the same set of 100 uniform simulated samples as described in Section 2.4.1, and calculate the median absolute proportion error (APE) as we did in Section 3.1. The results are summarized in Table S9, which shows that APEs are similar between the three X matrices. The result indicates that the performance using different simulation assumptions are largely the same.

**
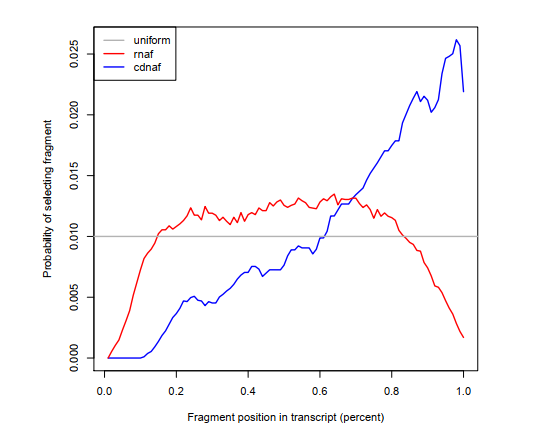
****Figure S12:** Positional bias models implemented in *Polyester* (PMID: 25926345). The grey line is the uniform read distribution, where reads are equally originating across the transcript. The red line refers to the rnaf bias, which will generate more fragments in the middle of a transcript than both ends. The blue line is the cdnaf bias, where a much higher coverage is in the 3’ end than the 5’ end.

**Table S9:** The median absolute proportion error (APE) using the three different versions of X: uniform X, biased X (cdnaf) and biased X (rnaf), for the quantification from the uniform simulated samples.

|  | Singletons | Non-paralogs | Paralogs |
| --- | --- | --- | --- |
| **Uniform X** | **0** | **0.18** | **0.12** |
| **Biased X**  **(rnaf)** | **0** | **0.16** | **0.10** |
| **Biased X**  **(cdnaf)** | **0** | **0.17** | **0.14** |
